# Effect of Ageing on the Tracking Characteristics of High-Temperature Vulcanized Silicone Rubber Hybrid Composites for High Voltage Insulation

**DOI:** 10.3390/ma13102242

**Published:** 2020-05-13

**Authors:** Mohamed Ghouse Shaik, Vijayarekha Karuppaiyan

**Affiliations:** Department of Electrical and Electronics Engineering, Shanmuga Arts, Science, Technology and Research Academy (SASTRA) Deemed University, Thanjavur 613403, India; vijayarekha@eee.sastra.edu

**Keywords:** polymeric hybrid composite insulation, ageing, hydrophobicity, tracking, dry band arcing, leakage current, surface degradation

## Abstract

Micro-sized aluminum trihydrate (ATH) filled silicone rubber is useful as insulation in the electric power system. The addition of nanofillers can improve further, its overall performance. However, the degradation of such silicone insulator due to ageing has not yet been thoroughly analysed. Motivated by this, an investigation was done to observe the effect of ageing on the tracking, and the material characteristics of SiO_2_ nanofillers added micro-sized ATH-filled Silicone rubber. For this, the samples were prepared using different weight percentage of SiO_2_ nanofillers and were thermally-aged and water-aged in the laboratory environment. A well-regulated tracking-test setup was assembled, and the leakage current characteristics of the fresh, thermal-aged and water-aged samples were observed, as per international electro technical commission standard (IEC) 60,587. After tracking, the surface morphology of these samples was studied using scanning electron microscopy (SEM). Further, energy dispersive X-ray analysis (EDAX) was carried out to observe the elements present at the surface and, Fourier transform infra-red (FTIR) spectroscopy was conducted to study the changes in the chemical structure. Investigations through the leakage current, SEM, EDAX and FTIR revealed that the addition of nanofillers improved the tracking characteristics of the aged hybrid composite insulation samples, thereby minimising any early failures.

## 1. Introduction

Composite polymeric insulators are emerging in the transmission and distribution sector because of its superior qualities compared to conventional ceramic and glass insulators. They possess significant advantages, such as being lighter, more comfortable transport, excellent hydrophobic property and high resistance to tracking and erosion [1]. However, the major concern is its long-term service performance, especially under a polluted environment. Due to the effects of pollution, different weathering condition and ageing, the composite polymeric insulator is susceptible to electro-thermal failure [2]. The composite polymeric insulator is made up of three components. The central core material is made up of fibre reinforced plastic (FRP), end metal fittings, and most importantly, the housing material made up polymers, such as polydimethylsiloxane (PDMS), the most commonly used, and other polymers such as ethylene propylene diene monomer (EPDM) and ethylene-propylene monomer (EPM) [2,3,4,5,6]. High-temperature vulcanised (HTV) silicone rubber (SiR) having base polymer as PDMS is the most commonly used housing material in the outdoor transmission tower composite insulators [2,3].

HTV-SiR is classified into two types, liquid type SiR (LSR) and millable-type SiR. Millable-type SiR is also known as high consistency SiR (HCR). To improve the performance of SiR, micro fillers are generally added in the base polymer [2,3,4]. In HTV silicone rubber hydroxide fillers, such as micro-sized aluminum trihydrate (ATH) were added during the manufacturing stage to improve the thermal stability of the silicone rubber [2,3,4]. ATH fillers release water during the thermal heating of SiR and results in heat quenching [4].Between 30% to 40% of micron-sized ATH fillers are added to the base polymer so that the released water during the ATH exposure to high-temperature aids in removing heat from the SiR material [4]. However, the increased use of ATH filler concentration may result in a reduction in the tensile strength and elongation. Also, it increases the surface roughness during operating conditions due to the release of the hydrate [2,3,4].

The polymeric housing materials degrade due to environmental ageing, such as UV radiation, pollution, rain and heat [2]. Due to ageing, the material loses its hydrophobicity, which is the ability of the material to repel water. Although, polymeric insulators have excellent hydrophobic recovery property, the temporary loss of hydrophobicity, due to ageing, may result in a frequent flashover under fog/mist conditions. During this condition, a considerable amount of leakage current will flow through the surface of the material and create dry band arcing. Dry ban arcing cause ohmic heating of the insulation surface and erode the surface of the material [1,2,3,4]. To understand the mechanism of degradation under various environmental ageing conditions, many methods have been proposed. The accelerated ageing studies of polymeric insulators were performed in the laboratories mainly to understand the resistance to electrical tracking, its thermal stability and the changes in the hydrophobic property of the samples [7,8,9,10,11,12,13].

Different methods have been proposed by the researchers, to improve tracking and erosion resistance of polymeric insulators. Recently, Nazir et al. [7] studied the effect of tracking and erosion resistance in room temperature vulcanised (RTV) Silicone rubber by adding micro-ATH/AlN/BN particles with different weight percentage. Verma et al. [9,10] investigated the tracking resistance effect of micro-ATH-filled liquid silicone rubber (LSR) and high-temperature vulcanised (HTV) silicone rubber under acid rain condition.

Haddad et al. [14] proposed textured liquid silicone rubber insulator design, which improves tracking resistance by increasing the creepage distance and reduces leakage current magnitude and electric field strength. Waters et al. [15] studied the dry band discharges in the textured polluted polymeric insulators and studied the improved tracking performance. Slama et al. [16], studied the thermal effects of dry bands and the associated discharge activity, in order to estimate the dynamic of the sparks in the textured polymeric insulators.

Due to the development of nanotechnology and the nanoparticle preparation methodologies, nano-sized fillers can be added in the polymer matrix to improve the thermal, mechanical and electrical properties. The nanoparticles have a larger surface area compared to microparticles. The nanocomposites offer better properties due to the interaction of the nanoparticles and the base polymer [17]. Adding different nanofillers in silicone rubber matrix improves the tensile strength, tear and abrasion resistance. The performance of filler in rubber is characterised by particle size and particle clusters, mass, level of interactions with rubber matrix, particle shape and surface property [18]. If the area of contact between fillers and rubber is increased, it leads to eminent reinforcement. The filler particle size, volume fraction, controls the surface contact area. The vital factor in determining the level of reinforcement is the extent of bonding between the rubber matrix and nanofillers [19].

Liu et al. analysed the surface arc discharges in room temperature vulcanized SiR/SiO_2_ nanocomposites and studied that the addition of nano SiO_2_ results in improved surface resistance to damage [20]. Du, B. X., and Li, Z. L. investigated the effects of the fluorination time, and the surface charges behaviour of SiR nanocomposites and reported the corresponding improvement in the dc flashover voltages [21]. Nazir et al. investigated to understand the addition of nano/micro SiO_2_ particles in enhancing the UV weathering resistance of RTV silicone rubber. Nazir et al. also studied the effect of different micro-fillers, like ATH, aluminum nitride (AlN) and boron nitride (BN) in improving the tracking and erosion properties of silicone rubber [22,23]. Guo, Y. et al. fabricated silicone rubber with platinum/amino-silane, which enhances the tracking resistance during dry band arcing period [24]. Khan, H. et al. investigated the effect of ATH-SiO_2_ reinforced RTV silicone rubbers in improving the tracking and erosion resistance along with water absorption resistance properties [25,26]. Jeon, Y. et al. studied the effect of different filler concentration on RTV silicone rubber on improving the tracking resistance, and they found that the tracking performance is similar to 20% by weight SiO_2_/SiR micro composites can be achieved with 5% by weight ATH/SiR nanocomposites [27].

All the recent literature studies have investigated the role of nanofillers/micro fillers in enhancing the tracking and erosion resistance during dry band arcing. But only very few researchers have studied the role of environmental effects on nanocomposites insulations. Loganathan et al. investigated the performance of thermal aged nano SiO_2_ filled silicone rubber and showed that the improvement of erosion resistance was achieved by adding nanofillers [28]. Suchitra, M. et al. investigated the hydrophobic nature of water aged epoxy nanocomposites samples. They concluded that the addition of nano and micro fillers improve the tracking and erosion resistance [29]. Zhang, H. et al. fabricated SiR nanocomposites using surface-modified TiO_2_ nanoparticles and studied the UV ageing behavior [30]. Rashid, A. et al. fabricated SiR/silica hybrid composites using SiO_2_ nanoparticles and microparticles. They investigated the environmental effects using the accelerated ageing test in a multi stress environmental chamber and, found that the hybrid composites offer high resistance against degradation due to ageing [31,32]. Techniques like scanning electron microscopy (SEM), energy dispersive X-ray analysis (EDAX), thermal gravimetric analysis (TGA), Fourier-transform infrared (FTIR) spectroscopy and differential scanning calorimetry (DSC) were used by the researchers to understand the material degradation characteristics due to ageing [28,29,30,31,32].

The resistance against different environmental stresses, such as rain, heat, UV radiation can be achieved by incorporating micro fillers and nanofillers in the base polymer matrix [31,32]. As of now, the environmental ageing effects on such high temperature vulcanised silicone rubber hybrid composites are unpredictable and still a research gap to be addressed. Laboratory ageing procedures can be used to simulate the effects of various environmental factors such asthermal ageing, UV ageing and water ageing and, the effect of such ageing on the tracking characteristics can be investigated. This work reports our experimental investigation, with a motivation to address the existing research gap. In essence, the effect of the ageing on micro-sized ATH and nano-sized SiO_2_ filled silicone rubber hybrid insulation. In this study, high temperature vulcanised silicone rubber hybrid-composite samples were first, prepared and subjected to water ageing and thermal ageing, as per the recommendations available in the literature [28,29]. After ageing, the tracking studies were conducted using an indigenously assembled well-regulated tracking test setup, as per the standards IEC 60,587 and ASTM D2303-13 [33,34]. The effects of ageing on this nano filled hybrid composite samples were investigated through leakage current measurement, contact angle measurement, SEM-EDAX analysis and FTIR spectroscopy, to obtain an insight into the influence of ageing on their tracking and material degradation characteristics.

## 2. Methodology

### 2.1. Sample Preparation

The base material for the preparation of HTV silicone rubber was Wacker-POWERSIL-210 (Munich, Germany), which is used by the manufacturers to develop outdoor polymeric insulators, such as composite insulators, arrestor bushings etc. The raw polymer used in the HTV rubber is polydimethylsiloxane (PDMS). The main chain of PDMS consists of silicon and oxygen atoms, and the methyl group forms the side chain, which is connected to silicon atoms. Micro-sized ATH fillers were initially added in the order of 35–40% by weight with PDMS during the preparation of base material.

Nanometric sized SiO_2_ nanoparticles with size 70 nm to 80 nm were used in this work. They were kept in a thermal oven at 100 °C for 24 h to remove moisture from the nanofillers and to make the surface of the particles dry [7,35]. HTV silicone rubbers are commercially manufactured with the help of two roll mill and injection moulding methods. During the manufacturing stage, when nanofillers are added, agglomeration of nanoparticles is inevitable. In this work, the surface of the nanoparticle was modified by mixing with ethanol, which acts as a surfactant. The main advantage to use ethanol as a surfactant is it changes the hydrophilic silica nanoparticles into hydrophobic, which makes it compatible with the hydrophobic polymeric chain. A magnetic stirrer was used to mix the ethanol surfactant and SiO_2_ nanoparticles.

Two roll mill technique was used to prepare the silicone rubber nanocomposite sheets. Initially, the gap between the rollers was kept to a minimum value of 1 mm. The temperature of the roller was set to 40 °C. The silicone rubber raw material was initially flattened between the rollers. The silicone rubber was masticated initially, and then slowly, the surface-treated nanoparticles-ethanol solution was mixed. When the degree of flattening with curing agent was achieved appropriately, nanofillers of 5%, 10% and 15% concentration by weight were added. The nanofiller added ethanol solution was gently added at the flat surface of the rubber and then the silicone rubber was again allowed to the two roll mill. Then the gap distance between the rollers was increased to 3 mm. The material was again flattened between the rollers followed by the addition of nanofillers. This ensures uniform dispersion of nanofillers in the polymer matrix during the two roll mill process. Peroxide curing is carried out to harden the silicone rubber samples. Peroxide curing of silicone rubber is also called vulcanisation. Dicumyl peroxide (C_18_H_22_O_2_) curing agent was added for curing the prepared samples [36]. Dicumyl peroxide is also known as Diisopropylbenzene peroxide orBis(α,α-dimethylbenzyl) peroxide. In the procuring phase, the samples were cured at a temperature of 180 °C for 20 min. In the post-curing stage, the samples were cured at a temperature of 200 °C for 4 h. The agglomeration effect was further studied using SEM analysis (model VEGA3-TESCAN, Brno, CZ).

**Figure 1:**
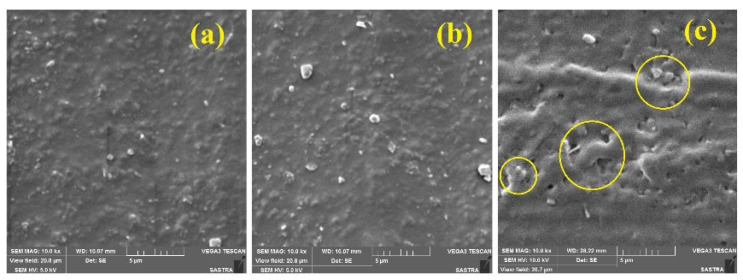
Scanning electron microscope images of (**a**) 5%; (**b**) 10%; (**c**) 15% Nano SiO_2_ filled silicone rubber.

SEM analysis was used to ensure uniform dispersion of nanoparticles in the silicone rubber matrix. The SEM images of nanoparticles filled silicone rubber samples are shown in Figure 1. It shows the change in morphology of the rubber sample for different concentration of nanofillers. It was observed that for 5% and 10% by weight filled nano SiO_2_ samples, the dispersion of fillers was in nanometric scale. But it was found that for higher filler concentration, i.e., 15% by weight, micro-sized agglomerated nanoparticles were observed. The circles in Figure 1c indicate the micro-sized agglomeration of SiO_2_ nanoparticles. Hence it is evident from the SEM analysis that using two roll mill technique the samples with lower concentration can be fabricated without much agglomeration of nanoparticles. In contrast, with higher nanofiller concentration, it was challenging to prepare samples without micro-sized agglomeration of nanoparticles. Hence in this work, 10% by weight filled samples were used for tracking studies.

The HTV silicone rubber material that was prepared has two interfaces. One interface is between the micro-sized ATH fillers and PDMS, and the other is between the nano-sized SiO_2_ filler and PDMS matrix. On the surface of the nano-SiO_2_, the leading active group is the hydroxyl group. The hydroxyl group forms the hydrogen bond with the PDMS backbone. Both PDMS and SiO_2_ has Si-O bond, part of PDMS backbone is arranged around the nano-SiO_2_. Due to this, the nano-SiO_2_ combines well with the PDMS matrix. For our study, the samples were cut into required dimensions of 120 × 50 × 3 mm. Fifteen such hybrid composite samples were used for tracking studies: Five each, in the fresh category, the thermally aged category and, the water aged category.

The ageing incorporated in our study is water ageing and thermal ageing. For the thermal ageing, five hybrid composite silicone rubber samples of dimension 120 × 50 × 3 mm were kept in an air circulated type, digital temperature indicator-cum-controller type hot air oven (Technico-laboratory model, Tamilnadu, India). The temperature accuracy of the hot air oven is ±2 °C. Thermal stress was maintained at a high temperature of 150 °C for a cumulative period of 240 h. Similarly, for water ageing, five samples were immersed in 500 mL of demineralised water for 500 h. The temperature of the water was kept at room temperature at atmospheric pressure [28,29]. In our previous research, a similar ageing methodology was adapted to study the effect of the ageing on Miro ATH filled Silicone rubber insulators [37].

A contaminant solution was prepared to simulate the deposition of a contaminant over the surface of the insulator as recommended by the standard IEC 60,587 [33]. NH_4_Cl solution with the conductivity of 2.5 mS/cm as recommended by the standard was used in this study [33]. Systronics digital conductivity meter was used to measure the conductivity. To prepare the contaminant, 0.1% ± 0.002% by mass of NH_4_Cl (ammonium chloride) analytical quality and 0.02% ± 0.002% by weight of iso-octyl-phenoxy-poly-ethoxy-ethanol (a non-ionic wetting agent) also known as Triton X-100 was added in 1 litre of de-ionized water [33]. Shimadzu AY220 digital weighing (Kyoto, Japan) scale with 0.1 mg accuracy was used to measure the weight of ammonium chloride and iso-octyl-phenoxy-poly-ethoxy-ethanol.

### 2.2. Experimental Arrangement

**Figure 2:**
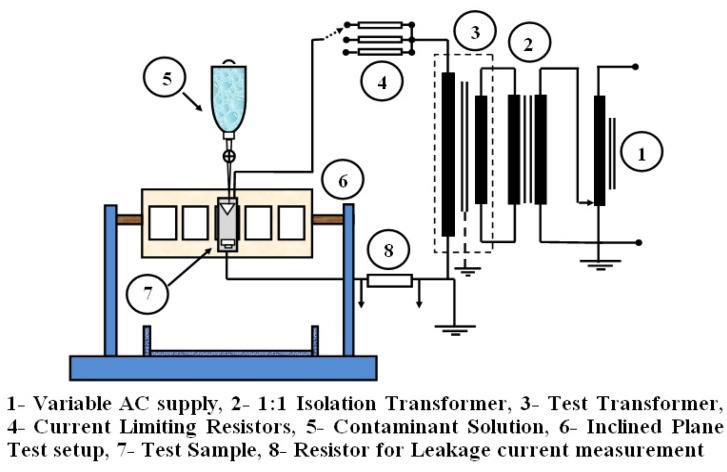
Experimental setup for the inclined plane test.

Single-phase AC supply 230 V, 50 Hz was applied to single-phase variac of rating 240 V/(0–270)V and the output of the variac was connected to the 1:1 isolation transformer. The output of the isolation transformer was connected to the 5 kV, 500 VA high voltage test transformer. A series current limiting resistance of 10 kΩ/22 kΩ/33 kΩ was connected to the secondary of the test transformer, and the other end of the resistance is connected to the HV electrode. The LV electrode is connected to a 25 W, 500 Ω resistance across which the leakage current is measured, and the respective waveforms are recorded in digital storage oscilloscope (DSO) (Keysight make DSO: Model no-DSO X1102, CA, USA). Figure 2 shows the experimental arrangement of the work.

**Figure 3:**
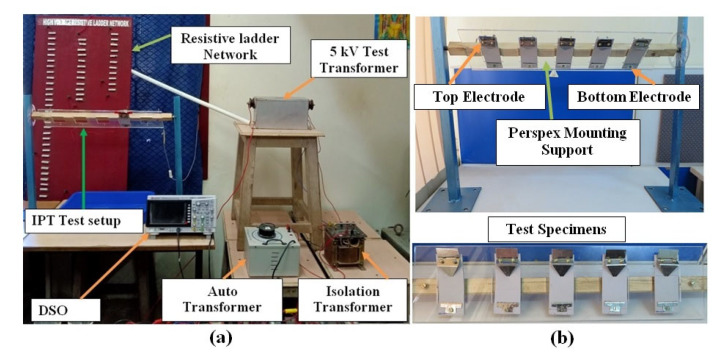
Photograph of the experimental setup and mounting support: (**a**) Experimental set up, (**b**) mounting support.

The contaminant solution NH_4_Cl was kept in an intravenous (IV) drip system, and the drops were allowed to drip on the filter papers. IEC 60,587 recommends testing five samples at a time. But due to the source rating limitations, one sample at a time was tested. The test samples are cleaned with isopropyl alcohol before use. The sample was mounted at an inclined angle of 45° in the mounting support. The IV tube carrying the contaminant solution is inserted between the layers of the filter paper, and the flow rate was adjusted to 0.3 mL/min as per IEC 60,587 standard which is equivalent to 5 drops per minute [33]. Figure 3 shows the photograph of the experimental arrangement.

The drops per minute were adjusted carefully to achieve 0.3 mL/min. A 4.5 kV of AC supply was applied across the electrodes and series resistance of 33 kΩ is connected as recommended in IEC 60,587 [33]. The primary voltage of 204 V is set to get 4.5 kV at the secondary side of the test transformer. The design of this low-cost indigenous tracking setup was reported in our previous tracking studies on micro ATH filled silicone rubber insulation [37].

### 2.3. Research Design

The research problem is mainly to investigate the effect of ageing on the ‘tracking and the surface degradation characteristics’ of the nanofillers, and micro-filler added hybrid composite high temperature vulcanised silicone rubber samples. Prior to conducting the electrical tracking test, the hydrophobicity of fresh, thermal-aged and water-aged samples was measured using contact angle measurement, to gain insight into the influence of ageing on the hydrophobicity of hybrid composite HTV silicone rubber.

Then the samples were subjected to tracking studies as per IEC6587 standard, to understand the variations in the leakage current magnitude for fresh and aged samples [33].

After 6 h of tracking test, the samples were removed carefully, and the highly eroded samples from each of the fresh, thermal-aged and water- aged sample groups were used for surface degradation studies. After electrical tracking of hybrid composites, SEM-EDAX and FTIR spectroscopy was carried out to analyse changes in the physical structure of samples [28,29,30,31,32].

## 3. Results and Discussions

### 3.1. Effect of Ageing on the Percentage Weight Change, the Contact Angle and Hydrophobicity

The hydrophobic property is considerably affected by thermal ageing, as the surface free- energy decreases after giving thermal stress at a temperature of 150 °C [28]. The mechanical strength of the sample reduces due to the dissolved low molecular weight components in the thermally aged samples [28].

The speed and quantity of the transfer of the low molecular weight components determine the change in the hydrophobicity of silicone rubber [29]. When the silicone rubber samples are continuously immersed in water to simulate the water-ageing effect, there can be an increase in weight of the sample because the water molecules are absorbed after water ageing. The change in positions of the methyl group will occur due to water ageing [29].

After ageing, there was a considerable change in weight of the samples due to depolymerisation, reorientation, and loss of low molecular weight components inside the sample. Table 1 shows the average weight of the samples and the standard deviation (SD) before, and after, ageing. Weight gain was observed in water aged samples, and weight loss was observed in thermal-aged samples. The overall percentage of weight gain and weight loss is shown in Table 1.

To investigate the hydrophobicity of the test samples, the contact angle was measured using the goniometer (Rame hart, NJ, USA). Hydrophobicity depends on various features like droplet size, volume, roughness and robustness of the sample surface. In this method, the water drop was made to fall on the silicone rubber sample surface. The contact angle between the drop and the surface was measured.The contact angle greater than 90° indicated good hydrophobicity and, less than 90° indicated that the sample had become hydrophilic [22,23,24,25,26].

**Figure 4:**
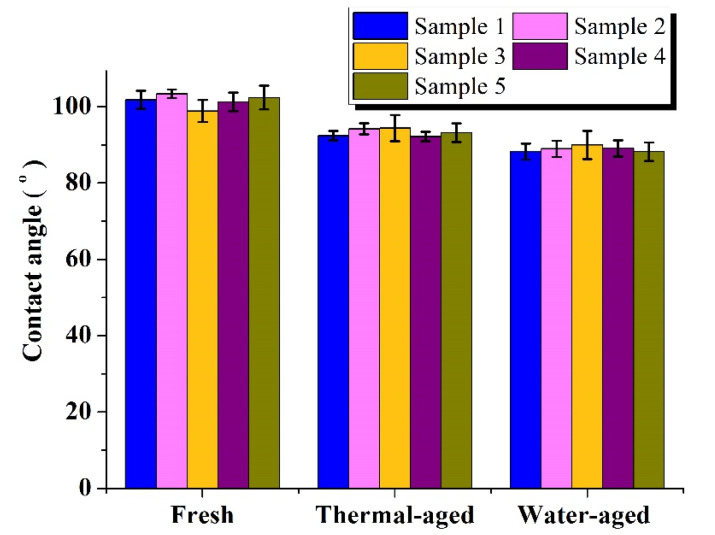
Contact angles of fresh, thermal- and water-aged samples.

Figure 4 shows that the hydrophobicity of the thermally aged and the water-aged samples decreased due to the effect of ageing. For every sample, the given contact angle is the average of eight measurements carried out at different locations of the sample. For fresh hybrid composites samples, the average contact angle was 101.56°. In contrast, for thermally aged samples, the average contact angle was reduced to 93.28°, and for water aged samples, the contact angle was further reduced to 88.91°.

### 3.2. Analysis of Leakage Current Variations During Tracking

**Figure 5:**
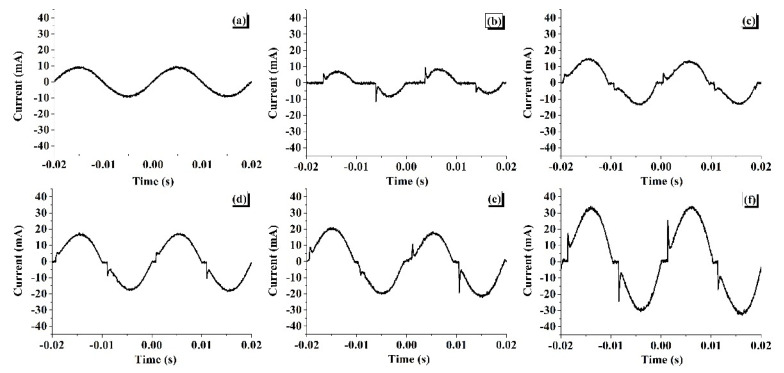
Variation of instantaneous leakage current of the fresh sample at different stages: (**a**) initial period, (**b**) dry band arcing period, (**c**) and (**d**) continuous arcing period, (**e**) hot spot initiation period, (**f**) carbonisation period.

Figure 5 shows the variation of instantaneous leakage current patterns recorded during the tracking of a fresh sample at different time intervals throughout the tracking test duration. Figure 5a shows the initial leakage current during the starting period of the test. It was minimum and continuous. Later, due to leakage current, the wet surface evaporated, and dry band regions were formed. Figure 5b shows the typical current pattern during the dry band arcing period. In this case, the current was discontinuous. Due to continuous rewetting and the dry band arcing the leakage current magnitude was increasing, as shown in Figure 5c,d. As time progressed, the magnitude of leakage current was raised, and a hotspot was observed near the bottom electrode. Figure 5e shows the leakage current pattern recorded during the initiation of the hotspot. With time, the erosion process was aggravated with carbonisation at the bottom region of the sample. Continuous arcing was observed during this period, and the leakage current pattern was as shown in Figure 5f.

During tracking experimentation, the leakage current patterns were almost similar for the fresh, thermal-aged and water-aged samples. However, it was observed that the magnitude of leakage current was different for the fresh and the aged samples.

**Figure 6:**
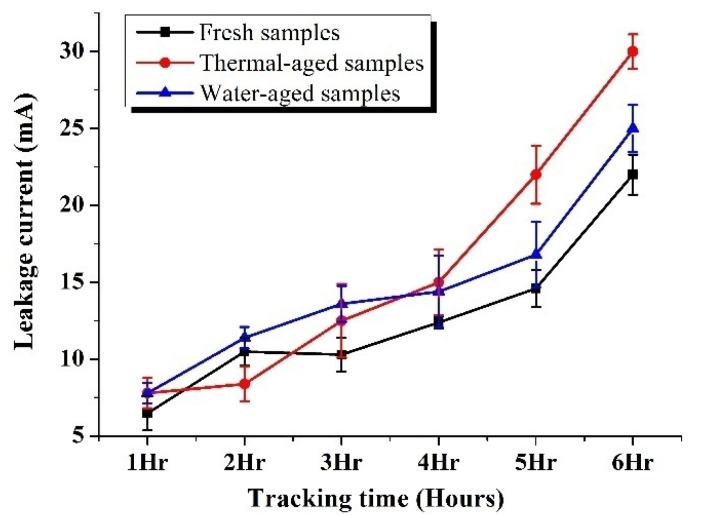
Variation of leakage current of fresh, thermal-aged and water-aged samples during tracking.

Figure 6 shows the variation of the root mean square (RMS) values of the leakage current (LC) of the fresh and aged samples during the tracking test. The instantaneous leakage current patterns and the RMS values were noted continuously, in the DSO. For the calculation purpose, the RMS values of leakage currents were noted at the end of each hour, ten times (last ten frames of the LC waveform, with two cycles covered per frame). Similar observations and calculations were made for all the five samples of a particular category (five samples each per the fresh, the thermal aged and the water aged category). Thus for each sample category, 50 RMS values were noted at the end of each hour. Then the mean and standard deviation were calculated for every hour. The mean RMS values were considered to represent the variation of leakage current against the time.

For fresh samples, the RMS values of leakage current (LC) was very minimum during the initial period of the test. It slowly increased after 4 h, and after that, the increase in current was almost linear. However, for thermal-aged samples, the current was almost constant during the initial period. After 2 h, a higher magnitude of current was observed compared to fresh samples. For water aged samples, initially higher leakage currents were observed due to the low value of hydrophobicity. However, from the third hour to the fourth hour, the current value was steady and almost constant. After 4 h, the current increase linearly. During the end of the test, the LC magnitude was highest for thermal-aged samples compared to the fresh and the water-aged samples. But up to 4 h of the test period, all the samples showed almost similar LC patterns, and the LC magnitude variations were minimum among the fresh and the aged samples. This could be due to the effect of the addition of nanofillers. It is reported in recent literature that the addition of a small amount of nanofiller improves the tracking resistance of polymeric insulations [25,26,27,28].

### 3.3. Analysis of Tracking Length and Loss of Weight after Tracking

**Figure 7:**
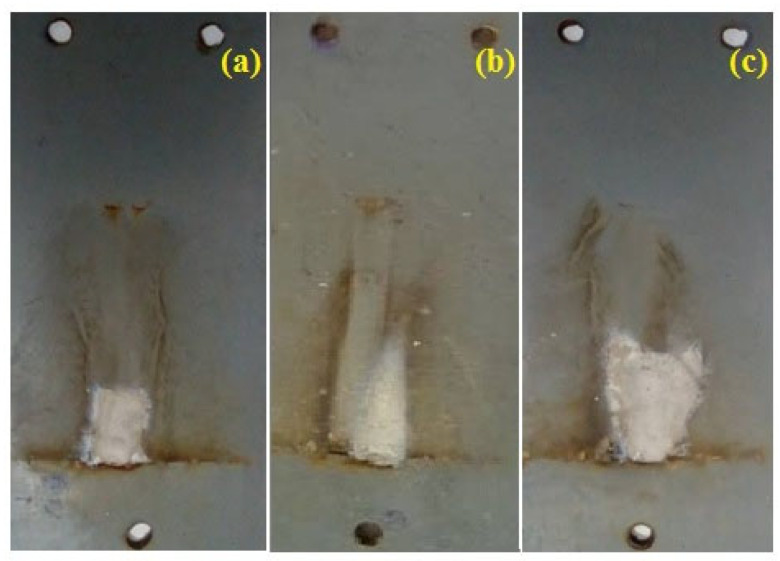
Images of the highly eroded and tracked samples: (**a**) Fresh, (**b**) thermal-aged, (**c**) water-aged.

After tracking tests, the tracked specimens were removed carefully. Figure 7 shows the images of such highly eroded and the tracked samples from the fresh, thermal-aged and water-aged groups.

The tracking length of individual sample was measured, and the average tracking length was computed. The samples were weighed before and after tracking, and the percentage weight loss due to tracking was calculated. Table 2 illustrates the comparison of the average tracking length and the average weight loss for all of the samples.

The average tracking length and average percentage weight loss of the thermal-aged and water-aged samples were comparatively higher than those of the fresh samples. The difference in the average weight loss between thermal aged and water aged sample was minimum. This ensures that the addition of nanofillers improves the tracking resistance performance of the hybrid composite samples even after ageing.

### 3.4. Morphological Studies Using Scanning Electron Microscopy

**Figure 8:**
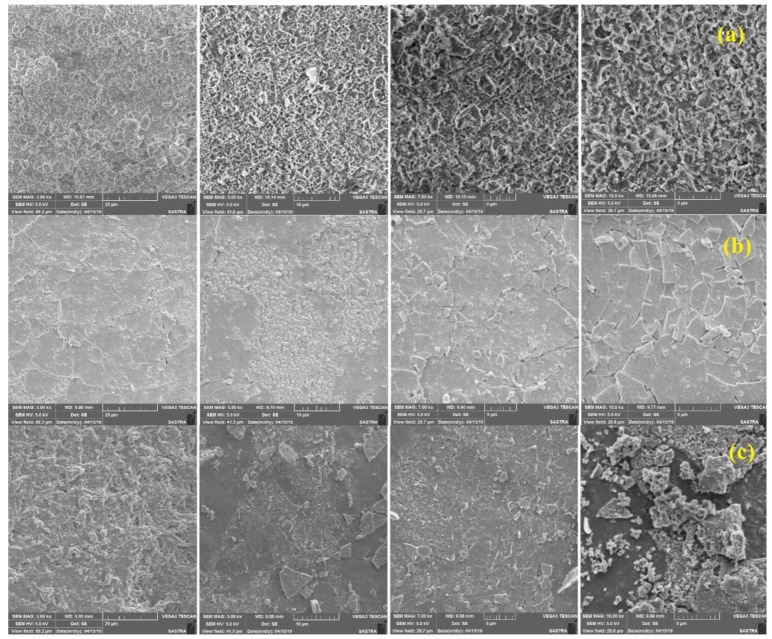
SEM micrographs of the eroded samples after the tracking with the magnification level of 3000, 5000, 7000, 10,000×: (**a**) Fresh, (**b**) thermal-aged, (**c**) water-aged.

SEM analysis was carried out to study the surface roughness, cracks, and surface morphology after tracking, and to understand the extent of degradation of the samples. ImageJ software was used to carry out the surface morphological studies. SEM images of the highly eroded region of the fresh, the thermal aged and the water-aged samples with a magnification level of 3000, 5000, 7000, 10,000× are shown in Figure 8. From the figures, it is clear that the surface morphology after tracking is different for the fresh sample and aged samples.

**Figure 9:**
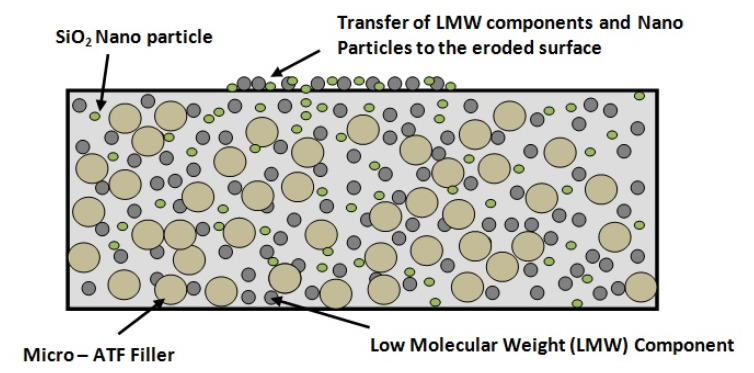
Schematic illustration of the transfer of the LMW components and the nanoparticles to the eroded surface.

At different magnification, distinct differences were observed at the surface of the tracked samples. Due to electrical tracking, oxidation at the surface of the silicone rubber occurs. Due to this, the hydrophobicity recovery process is retarded [28]. Figure 8a shows the SEM images of the fresh samples. The morphological changes in the highly eroded area were analysed. A rupturing pattern was observed with uniform degradation. The presence of voids and pits were found. This pattern was different in the thermal-aged and the water-aged samples. In the case of thermal-aged tracked samples, as shown in Figure 8b, uniform cracks and surface splitting was observed. Compared to the SEM images of the fresh samples, more surface cracks were found. This could be due to the loss of its hydrophobicity due to thermal ageing before electrical tracking [28]. After electrical tracking, the surface morphology was affected in the aged samples. In the case of water-aged samples, instead of the crack formation, more craggy irregular morphology was observed, as shown in Figure 8c. In the water-aged samples, the continuous steady leakage current was observed for a longer duration compared to fresh and thermal aged samples, as discussed in Section 3.2. Due to this continuous leakage current, ATH released water and alumina, which might have settled over the surface as residues. This may be the reason for the irregular shapes at the surface of the water-aged samples. Such irregular cracked surface may provide speedy transportation of the low molecular weight (LMW) components as reported in recent articles [7,23]. Figure 9 shows the schematic illustration of the transfer of the LMW components and the nanoparticles at the eroded surface.

Electrical tracking has altered the physical and chemical structure of the base polymer surface. Due to oxidation, the hydrophobic methyl group was changed to a hydrophilic hydroxyl group. However, after a certain time, the recovery of hydrophobicity was observed due to the reorientation of the LMW component from the bulk polymer matrix [7,23]. During this process, the nanoparticles were also transferred to the eroded surface. The migration of nanoparticles over the surface can effectively improve the tracking resistance of the nano filled composites. It is reported that the recovery of hydrophobicity is slower compared to the unfilled samples, because it offers resistance to the transport of the LMW from the bulk structure to the polymer surface [23]. The improved corona discharge activity due to nano alumina was recently reported in [23]. The formation of alumina oxide (Al_2_O_3_) as the decomposition of ATH fillers might have settled over the surface of the water aged sample. From this study, it is clear that the addition of nano and microparticles in the polymer matrix has significantly improved the tracking resistance, even after the thermal and water ageing.

### 3.5. Energy-Dispersive X-ray Analysis

**Figure 10:**
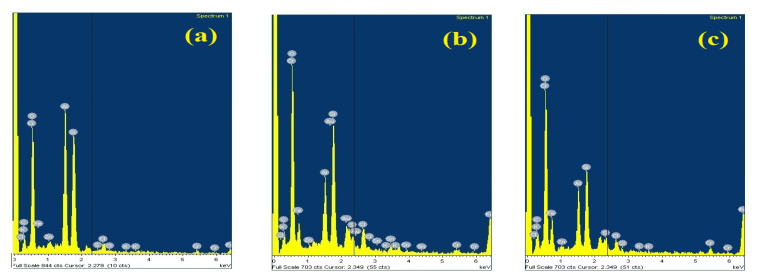
EDAX spectra of tracked samples: (**a**) Fresh, (**b**) thermal-aged, (**c**) water-aged.

EDAX was used in our study to know about the changes happening in the chemical composition of the tracked samples. Figure 10 shows the EDAX spectrum of the fresh, thermal-aged and water-aged samples after electrical tracking test. The results of EDAX will be in weight percentage (weight%) or atomic percentage (atomic%). Weight% is the function of the atomic weight of the elements present in the sample, whereas atomic% is the function of the number of atoms present in the sample [28].

The major elements C, O, Al and Si, were found on the surface of the fresh, thermal-aged and water-aged samples, as indicated from the EDAX spectrum. In Table 3, the comparison of both weight% and atomic% for different elements present in the fresh, thermal-aged and water-aged sample were presented. As atomic% gives the percentage of one kind of atom relative to the total number of atoms in the sample, the change in atomic% was analysed in detail. It was clear from the table that the atomic% of C had reduced and the atomic% of O had increased due to ageing. The reduction in atomic% in C and the increase in the atomic% of O were due to the deformation of Si-CH_3_ chain, which might have changed to Si-OH [28]. The elemental changes indicated that the aged samples degrade more compared to fresh samples.

A decrement in the atomic% of the aluminum was observed, due to ATH micro-fillers decomposition. An increase in the atomic% of Fe was found in the aged samples, which might have released due to the degradation of bottom electrodes due to a high magnitude of leakage current in the aged specimen. From the EDAX analysis, it was clear that several elemental changes had occurred in the aged samples. However, the atomic% change in the elemental composition was not too high. This could be the effect of high resistance against electrical tracking due to the addition of a small percentage of nanofillers in the base polymer matrix.

### 3.6. Fourier Transform Infrared (FTIR) Spectroscopy Analysis

**Figure 11:**
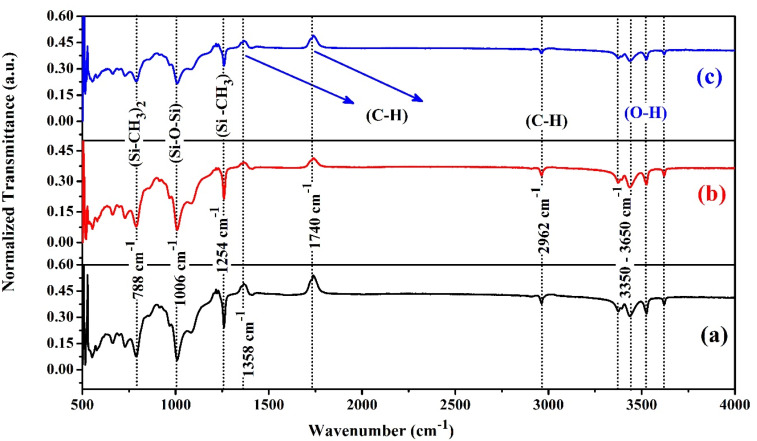
FTIR-Spectrum of the highly eroded and tracked samples: (**a**) Fresh, (**b**) Thermal-aged, (**c**) Water-aged.

The FTIR spectra of the fresh, thermal-aged and water-aged samples are shown in Figure 11. The obtained results were interpreted based on similar observations in our previous work and other literature [31,37]. The FTIR spectrum of the fresh tracked hybrid composite sample was shown in Figure 11a. This spectrum was used to compare the FTIR spectrum of the thermal aged and the water- aged samples, as shown in Figure 11b,c. For understanding the change in the transmittance value, all the patterns were normalised, and a stacked pattern was given for better clarity. In the FTIR spectrum, the region between 500 cm^−1^ and 1500 cm^−1^ is the fingerprint region and the region between 1500 cm^−1^ and 4000 cm^−1^ is the functional group region [37].

The dips observed in the transmittance plot at 3350–3650 cm^−1^ indicated the formation of the O-H hydroxyl bond. For the thermal-aged and the water-aged samples, similar dips were observed. There was not much difference in the transmittance spectrum in this region. Slightly higher transmittance value was found in the water aged sample, which indicated a higher degree of hydroxylation in the water aged sample. The higher transmittance was due to the dehydration of ATH fillers and the sustained- leakage current, as discussed in Section 3.2.

The dip observed at 2962 cm^−1^ indicated the decrease in the intensity of the C-H stretch in CH_3_ bond. This indicates C-H symmetric stretching, which affects the polymer chain. Due to this, shrinkage of the polymer chain occurs, and it hardens the surface of the polymeric sample. This results in less vibration of C-H bond, andconsequently, C-H stretch intensity decrease. In the aged sample, a similar dip was observed, but, the ageing did not affect the transmittance value much. The introduction of inorganic nanoparticles in the polymer matrix offered a better barrier against surface ageing [22,23,24,25]. This could be the reason for the similar transmittance pattern for the fresh and the aged samples. The thermal and water ageing did not influence much on the hybrid composite materials.

There was an increase in the transmittance spectrum at 1740 cm^−1^ and 1358 cm^−1^. This is due to the formation of carbonyl degradation products due to electrical tracking.

At 1254 and 788 cm^−1^, the changes in the transmittance peaks for the thermal-aged and water-aged sample were almost the same. There was not much change in the intensity of the Si-C bond. This is because the skeleton of the polymer matrix is made up of the Si-C Bond.

In the region 1006 cm^−1^, there was a difference in the peak intensity of the thermal-aged and the water-aged samples, due to Si-O stretch vibration. There was not much difference between the fresh and aged samples.

This indicated that the ageing of the polymeric hybrid composite samples was not affected much in tracking performance and its material degradation. Hence it is clear that the addition of a small percentage of SiO_2_ nanofillers has significantly improved the tracking resistance of polymeric materials.

## 4. Conclusions

Tracking and surface degradation studies were carried out on the fresh, thermally aged and water aged samples of nano-SiO_2_ filled silicone rubber. Silicone rubber samples were subjected to thermal and water ageing for a particular period, and the change in weight of the samples was analysed. The hydrophobic property of silicone rubber samples was investigated by sessile drop goniometer, and it was found that the hydrophobicity decreased after ageing.

The inclined plane test was conducted for fresh and aged samples at 4.5 kV (AC) with the contaminant solution as ammonium chloride at a flow rate of 0.3 mL/min as per IEC 60,587 standard. It was observed that the maximum magnitude of the leakage current was found in the thermal-aged samples. However, the differences in the variations in the magnitude up to 4 h were almost minimum. These minimum variations could be due to the presence of nanofillers in the polymer matrix, which offers higher tracking resistance. The percentage of weight loss after the erosion of each tracked sample was determined, and the average weight loss was calculated. The difference in the average weight loss between thermal aged and water aged sample was minimum. This ensures that the addition of nanofillers improves the tracking resistance performance of the hybrid composite samples even after ageing.

SEM analysis was carried out in order to understand the surface degradation mechanism of the fresh and aged samples after tracking. It was observed that the major and minor cracks were formed in the thermal-aged sample, and the craggy irregular structure was observed in water-aged samples. This indicates that the amount of leakage current and the duration of the higher magnitude current affects the surface morphology of the aged samples. To understand the change in the chemical composition, EDAX analysis was carried out. It was observed that atomic% of C reduced and atomic% of O increased due to ageing. FTIR analysis was carried out to analyse the hydroxyl group and methyl group reorientation. The formation of hydroxyl bond, C-H bond stretch, Si-O stretch, Si-Cstretch and carbonyl degradation products were studied. It was observed that the difference in the transmittance spectrum for fresh and aged samples were minimum. These minimum differences indicate that the addition of 10% by weight nanoparticles in the micro ATH filled silicone rubber composites dramatically improve the tracking resistance and surface degradation.

## Figures and Tables

**Table 1:** Comparison of % weight change due to ageing.

Water Aged (mg)		Thermal Aged (mg)	
Before Ageing	After Ageing	Before Ageing	After Ageing
Average weight 21,647.4	Average weight 22,816.4	Average weight 24,759.6	Average weight 24,737.3
Standard deviation 1123.3	Standard deviation 1187.5	Standard deviation 1326.7	Standard deviation 1275.4
% Weight gain = 5.4%		% Weight Loss = 0.09%	

**Table 2:** Comparison of tracking length and weight loss.

Sample Type	Average Tracking Length (cm)	Average Weight Loss (%)
Fresh samples	2.2	1.11
Thermal-aged samples	2.9	1.45
Water-aged samples	3.1	1.32

**Table 3:** Elemental composition analysis of the tracked samples-with EDAX.

Elements		Fresh	Thermal-Aged	Water-Aged
C	weight%	9.24	9.32	8.00
	atomic%	26.95	24.81	26.40
O	weight%	24.96	29.75	23.17
	atomic%	54.68	59.47	57.39
Al	weight%	6.90	2.82	2.81
	atomic%	8.96	3.34	4.13
Si	weight%	6.49	5.27	3.58
	atomic%	8.10	6.00	5.05
S	weight%	0.14	0.58	0.46
	atomic%	0.16	0.58	0.57
Cl	weight%	0.14	1.23	0.55
	atomic%	0.53	1.11	0.62
K	weight%	0.14	0.01	0.04
	atomic%	0.02	0.01	0.04
Cr	weight%	0.14	0.35	0.76
	atomic%	0.22	0.22	0.58
Fe	weight%	0.14	5.93	7.07
	atomic%	0.52	3.39	5.02
Zn	weight%	0.14	0.36	0.33
	atomic%	0.23	0.17	0.20
